# Investigations of Micro-Deformation in Monocrystalline Copper at Low Temperatures via Indentation

**DOI:** 10.3390/mi13071043

**Published:** 2022-06-30

**Authors:** Shunbo Wang, Dan Zhao, Yihan Niu, Zhaoxin Wang, Hongxiu Yang, Hongwei Zhao

**Affiliations:** 1 Key Laboratory of CNC Equipment Reliability, Ministry of Education, Changchun 130025, China; wangshunbo@jlu.edu.cn; 2 School of Mechanical and Aerospace Engineering, Jilin University, Changchun 130025, China; danzhao@jlu.edu.cn (D.Z.); niuyh20@mails.jlu.edu.cn (Y.N.); zhaoxinw19@mails.jlu.edu.cn (Z.W.); 3 Mechanical Engineering College, Beihua University, Jilin 132000, China

**Keywords:** monocrystalline copper, indentation, low temperature, plastic deformation, elastic recovery

## Abstract

Indentation experiments on differently oriented faces of monocrystalline copper were conducted to investigate the micro-deformation process at temperatures ranging from room temperature to 150 K. The morphologies and textures of the residual imprints were observed using electron microscopy. Distinct slip bands were observed inside the imprints at 150 K compared to smooth surfaces at room temperature. Molecular dynamics simulations were performed to identify the deformation process beneath the indentation region. The results showed that plastic deformation was inhibited with decreasing temperature, but elastic recovery during the unloading process was enhanced, resulting in inner slip bands (ISBs) being observable in the residual imprints. The performances of these ISBs were strongly associated with the angles between the indentation direction and major slip surfaces and could be considered microscopic forms on the surfaces of aggregated geometrically necessary dislocations (GNDs). This work helped reveal the micro-deformation mechanism of indentations inside imprints.

## 1. Introduction

The micro deformation response of a material’s surface at the micro- and nanoscale can be effectively investigated through indentation experiments [1,2]. Complex stress distributions, including those of the hydrostatic pressure and octahedral shear stress, occur during the indentation process. Monocrystalline materials are ideal for indentation research because of their simple and defect-free crystal structure [3]. Numerous investigations have been conducted on monocrystalline copper to investigate the elastic and plastic deformation of face-centered cubic (FCC) metals [4,5]. As the spherical or conical indentation tip penetrates the copper, several significant phenomena occur in the indentation curves and around the imprint, including pop-ins [6], an indentation size effect (ISE) [7], an anisotropic effect [8], and lattice rotations [9]. Simulations based on molecular dynamics (MD) provide a means of revealing the defect generation and extension under the indentation tip [10,11]. The characteristic phenomenon and evolution trend of the deformation are in good agreement with the experimental results. Because the deformation process beneath the indentation tip is difficult to observe using experimental methods, and it is difficult to form large-scale defects in simulations, a fundamental understanding of the surface deformation adaptation process is still unavailable.

Low temperatures are a common working condition for metals, especially in space. The plastic response must be carefully studied to ensure the reliable functioning of their structures. Because of instrument condition limitations, most low-temperature indentations (or hardness tests) are usually conducted at 77 K, with the material immersed in liquid nitrogen [12,13]. The deformation mechanisms involved in the relationship between the imprint formation and temperature decrease are difficult to determine at room temperature (RT) and the temperature of liquid nitrogen. Although the effect of grain boundaries on the indentation plastic zone has been well investigated for polycrystalline and nanocrystalline copper [14,15], little is known about the intracrystalline deformation features at low temperatures. Insight into these features is necessary to elucidate the correspondence between the contact plasticity and temperature variation at the micro- and nanoscale, which can contribute to a better understanding of tests in the general environment.

This work was initiated to systematically investigate the plastic response of monocrystalline copper, from the surface deformation phenomenon to the defect distribution beneath the indentation tip of imprints at various low temperatures. Our investigation assessed (i) the anisotropic behaviors under an indentation load across a series of temperature ranges and surface orientations, (ii) the occurrence of inner slip bands inside residual imprints and their evolution with decreasing temperature, and (iii) the geometric necessity of the inner slip band for the plastic deformation of a material in response to indentation. Along these lines, our work represents a systematic attempt to investigate the influence of temperature on the indentation response of monocrystalline copper and further understand the role of geometric characteristics within the indentation plastic zone.

## 2. Methods

### 2.1. Experimental

This study used high-purity (99.99%) monocrystalline copper samples with normal surfaces, *n* = (100), (110), and (111), which were provided by the MTI Corporation in Hefei, China. The thickness of the samples was approximately 1 mm, and the surface roughness of the polished side was less than 1 nm. This side was used for the indentation measurements. The crystal orientation of the specimen surface was carefully examined using electron backscattered diffraction (EBSD), and the tolerance was within ± 2°.

**Figure 1:**
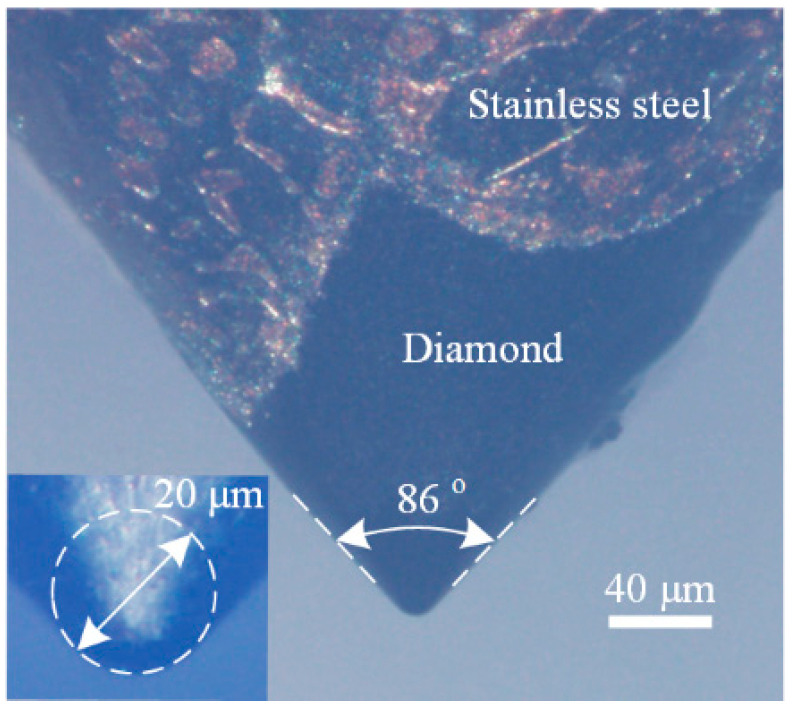
Indentation tip adopted in this work.

Indentation tests were performed using a customized cryogenic indentation device with a cryogenic modulus that utilized liquid nitrogen and a vacuum modulus to prevent the occurrence of ice [16]. A spheroconical indentation tip (Synton-MDP) with a radius of ~10 μm and cone angle of ~86° was adopted, as shown in Figure 1. Samples with (100), (110), and (111) crystal faces were indented at a constant loading/unloading rate of 10 mN/s and maximum indentation loads of 50–500 mN. The test temperatures were set to RT, 250, 200, and 150 K. Indentation was conducted thrice at each temperature and maximum load condition.

Scanning electron microscopy (SEM) measurements were performed using a JSM-6700F system. Two modes of observation, namely secondary and low secondary electron images (SEI and LEI, respectively), were used to emphasize different image details. Samples for cross-sectional transmission electron microscopy (XTEM) were prepared using an FEI Helios 600i focused ion beam system. The transmission electron microscopy (TEM) observations made in the current study were obtained using a Talos F200 instrument operated at an accelerating voltage of 200 kV.

### 2.2. MD Simulation Model

Two 40 nm × 36 nm × 40 nm monocrystalline copper atomic models with (110) and (111) crystal faces for the indentation surface were built. Each model contained approximately 4.8 million atoms, and the thicknesses of the boundary and thermostatic layers were 0.8 nm and 2 nm, respectively. Periodic boundary conditions were set in the x and z directions. The interaction between the copper atoms was described using the embedded atom method potential [17,18,19]. The model was relaxed in the canonical (NVT) ensemble for 20 ps before loading to obtain the equilibrium configuration at various temperatures. Thereafter, a virtual indenter with a radius of 8 nm was selected for indentation, which was initially located at 0.5 nm above the specimen surface. The indenter was indented into the specimen along the negative direction of the y-axis at a velocity of 50 m/s, and the maximum depth was 3 nm. Subsequently, the indenter returned to its initial position along the inverse direction at the same velocity to complete the indentation process. During the simulation, the integration time step was set at 1 fs. In this study, the indentation process was implemented using the atomic simulation software LAMMPS [20]. The calculation results were post-processed using the visualization software OVITO [21].

## 3. Results

**Figure 2:**
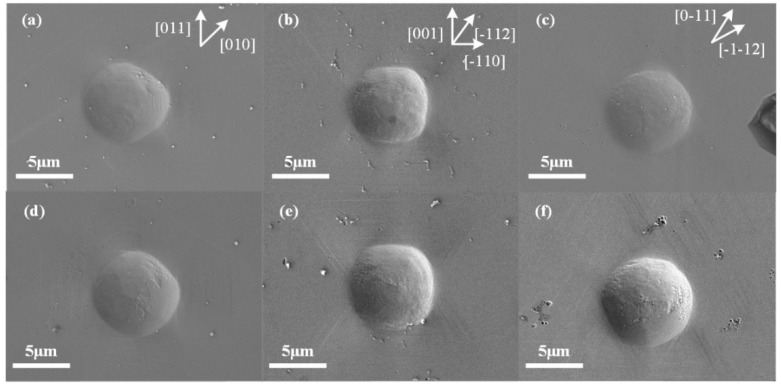
Residual imprints of (**a**,**d**) (100)-, (**b**,**e**) (110)-, and (**c**,**f**) (111)-oriented crystal surfaces after indentation with spheroconical tip at (**a**–**c**) 150 K and (**d**–**f**) RT with maximum load of 50 mN.

Figure 2a–f show the residual imprints on the (100), (110), and (111) crystal faces of monocrystalline copper with a maximum load of 50 mN at 150 K and RT. Because the spheroconical indentation tip was isotropic on the testing plane, the shape of the imprints indicated the anisotropic properties of the tested material. Numerous experimental and simulated investigations have presented the orientation dependence of the indentation anisotropy of copper at RT [22,23]. The occurrence of four-, two-, and three-fold symmetry for the (001)-, (011)-, and (111)-oriented crystals was due to the pile-up patterns on the surfaces. This phenomenon also occurred in the current work, as well as at a low temperature of 150 K. Minor changes occurred between 150 K and RT at a load level of 50 mN. The size of the imprints decreased, which was related to the change in hardness (see our previous research [16]). However, with the pile-up around, and slip bands outside, the imprint became difficult to observe at low temperatures on the (111)-oriented crystal.

**Figure 3:**
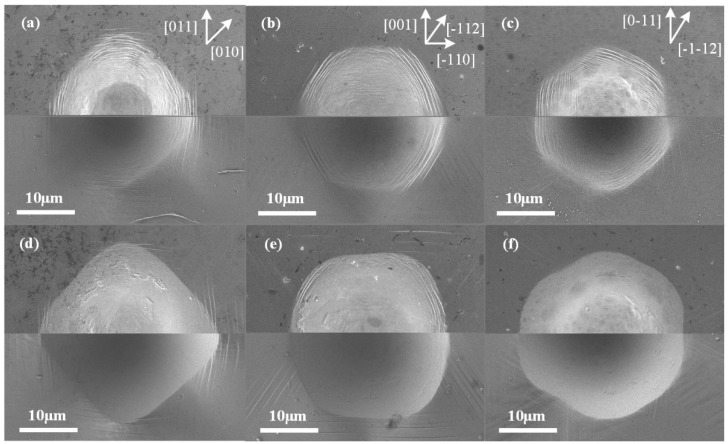
Residual imprints of (**a**,**d**) (100)-, (**b**,**e**) (110)-, and (**c**,**f**) (111)-oriented crystal surfaces after indentation with spheroconical tip at (**a**–**c**) 150 K and (**d**–**f**) RT with maximum load of 500 mN.

When the maximum indentation load increased to 500 mN, more significant in-plane anisotropy occurred in the residual imprints at RT, as shown in Figure 3d–e. Meanwhile, the surface inside the imprint, which was flattened under the high pressure applied via the indentation tip, was essentially smooth, presenting a common state for indentation tests (except for some composite materials). However, some interesting phenomena occurred at low temperatures inside the imprints, as shown in Figure 3a–c. The top half of each image was captured in the SEI mode to show the deformation details, while the bottom half was captured in the LEI mode to obtain more stereoscopic morphology information. One primary difference was that the low temperature led to slip bands inside the imprints, which we refer to as inner slip bands (ISBs). Meanwhile, the ISBs were much more significant than the common slip bands outside the imprints or outer slip bands (OSBs). The height of the ISBs was approximately 110–160 nm (as measured using subsequent XTEM results), which was much higher than that of common OSBs.

Compared with the results for the lower load in Figure 2, the phenomena of pile-up and OSBs became much weaker at low temperatures under 500 mN. Meanwhile, the boundaries of the imprints became discontinuous on the (110)- and (111)-oriented crystals, and formed two states: an indistinct boundary and an ISB boundary, which alternated in a complete imprint boundary, as shown in Figure 3a,c. Indistinct boundaries were generated in the [001] and [00 $\bar{1}$ ] directions on the (110)-oriented surface, whereas they were uniformly distributed in the [1 $\bar{2}$ 1], [11 $\bar{2}$ ], and [ $\bar{2}$ 11] directions on the (111)-oriented surface. It should be noted that the boundaries were concave in the corresponding positions at RT, as shown in Figure 3e,f. The remaining relatively distinct boundaries at low temperatures were distinguished from single-line boundaries at RT but consisted of existing ISBs.

The differences in the load conditions between the 50 mN and 500 mN contacts mainly consisted of the load value and contact status. According to the size of the indentation tip and residual imprints calculated using SEM images, the contact angle at the boundary region was ~23.5° for the 50 mN imprints, while the angle was ~56° for the 500 mN imprints. A lower total stress and higher contact angle inevitably led to a significant increase in the stress gradient. It could be inferred that the aforementioned phenomena of ISBs, weaker pile-ups, and discontinuous boundaries at higher indentation loads should have been induced by a stronger plastic deformation and elastic behavior during the entire indentation process, which will be discussed in the subsequent section.

## 4. Discussion

### 4.1. Plastic Response

**Figure 4:**
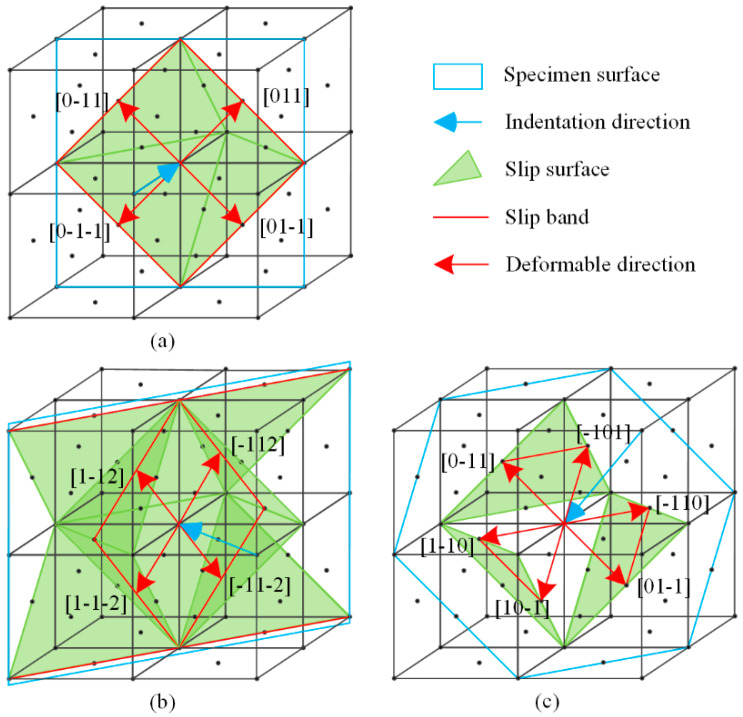
Crystallography of plastic deformation in (**a**) (100)-, (**b**) (110)-, and (**c**) (111)-oriented crystal surface indentations.

To better understand the plastic deformation mechanism in the FCC metal during the indentation process, Figure 4 shows the relationship between the indentation directions (blue arrows) involved in this study and the corresponding primary slip surfaces (green faces) according to the existing slip system theory. The direction of the OSBs could be easily determined by the intersection of the lines of the slip surfaces and specimen surfaces (blue wireframe). Because slip motion in FCC crystals can only be conducted along specific slip directions, <110> on slip surfaces, the deformable directions of the imprints performed on the indentation surfaces should be parallel to the existing OSBs. This can explain the distribution of OSBs and the shape of the residual imprints at RT very well. However, the formation mechanism for ISBs at low temperatures remains unclear.

**Figure 5:**
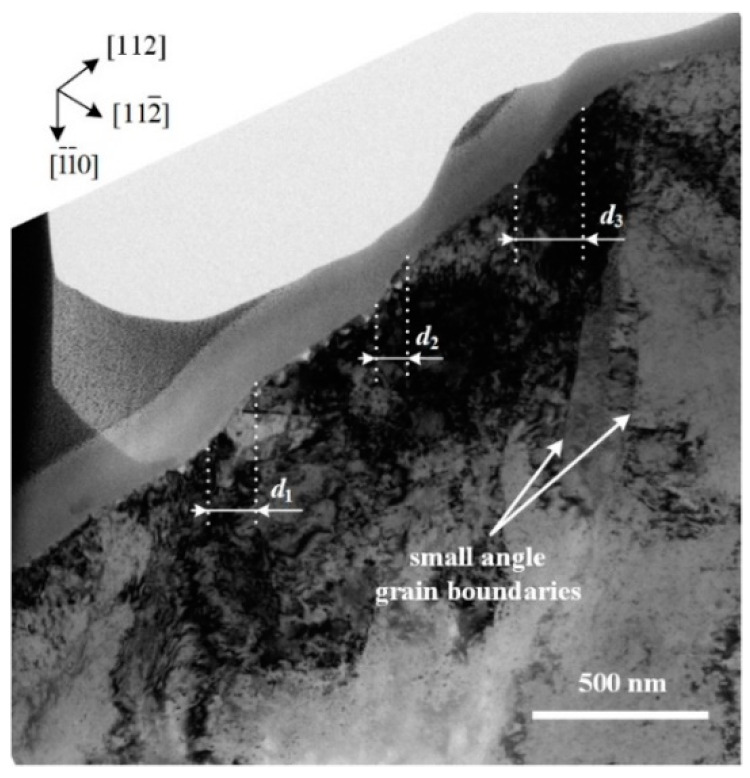
Bright-field XTEM image of ISBs formed with load of 350 mN at 150 K.

Therefore, the relationship between the ISBs and OSBs should be considered. Based on the distribution of the two types of slip bands in the (100)-oriented imprint (Figure 3a), a confusing conclusion may be obtained: the ISBs were converted from OSBs during the loading process with the imprint overlapping the existing OSBs. However, the (110)-oriented imprints (Figure 3b,e) provided a different perspective. The OSBs in the [ $\bar{1}$ 1 $\bar{2}$ ] direction (lower right part of each image) mainly consisted of slip bands along [1 $\bar{1}$ $\bar{2}$ ] and [ $\bar{1}$ 10], whereas the contiguous ISBs were along [ $\bar{1}$ 12]. This was essentially impossible in the conversion process. On the other hand, Figure 5 shows an XTEM image of the (110)-oriented indent with a cross-section along the (1 $\bar{1}$ 1) direction, indicating that the ISBs and OSBs had opposite step gradient directions. The generation of ISBs should have been independent and related to the local deformation inside the imprint.

**Figure 6:**
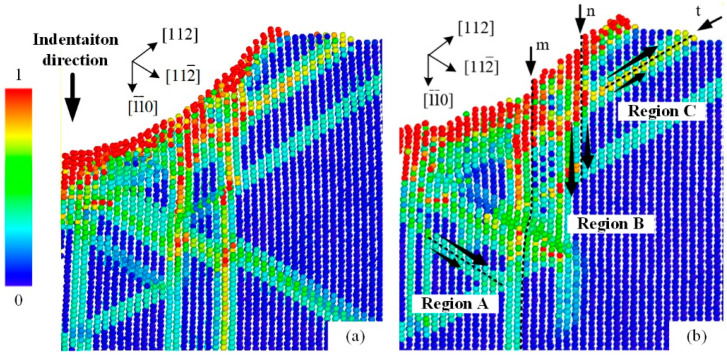
MD simulated shear strains and atomic arrangements in (110)-oriented indentation at 150 K: (**a**) at maximum indentation displacement, and (**b**) after unloading process.

Figure 6 shows the simulated shear strain distribution and atomic arrangement of the [110]-oriented indentation along the (1 $\bar{1}$ 1) slip surface at 150 K (the same as Figure 5) during the unloading process. The surface inside the indentation region fit well with the virtual indenter under the maximum displacement (Figure 6a). This is a common state during indentation, as any possible protruding region inside the imprints will endure much higher local pressure. As the indentation tip was separated from the contact surface (Figure 6b), two significant protruding parts (m and n) were produced by the elastic recovery process inside the imprint. Meanwhile, corresponding slip activations (dotted lines) occurred beneath the ISBs of m and n. Thus, we can preliminarily use the following process to describe the generation of ISBs. Because of the shear stress at the edge of the spherical indentation tip, slip motions with a downward deformation component occurred in the subsurface during the loading process and extended to the surface inside the imprint. In contrast to the free surface of the surrounding regions outside the imprints (pile-ups, marked as t), the discontinuities were squeezed and flattened under the tip surface. During the unloading process, the elastic energy stored inside the discontinuities was released, and the ISBs were produced.

**Figure 7:**
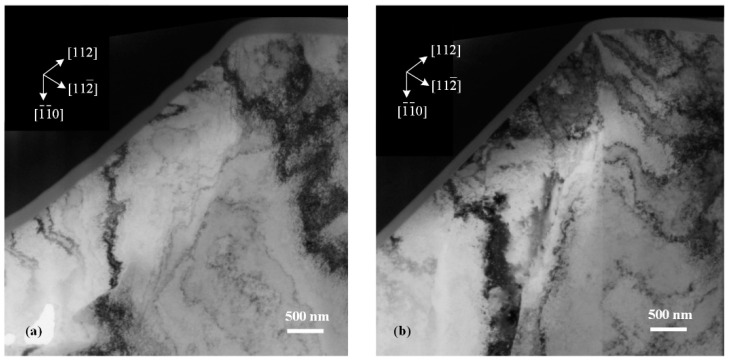
XTEM images of (110)-oriented imprints formed with load of 350 mN at (**a**) 150 K and (**b**) RT.

Because the ISBs and OSBs shared a single slip system for plastic deformation, the directions of these two types of slip bands on the specimen surface should have been the same. Meanwhile, the ISBs underwent further deformation to adapt to the shape of the indentation tip, inducing a bent state, especially in the (100)-oriented imprint. As for imprints conducted at RT, the elastic recovery was weaker than that at low temperature and not sufficient to generate obvious ISBs (the details of the elastic recovery will be discussed in the next section). However, why were ambiguous ISBs observed in the (110)-oriented imprint at RT (Figure 3e), and why were the ISBs the most obvious among the three oriented faces? We noted that the included angles between the indentation direction and the major slip surfaces were ~35.3°, 0°, and 18.4° for the (100)-, (110)-, and (111)-oriented surfaces, respectively, which could be calculated using Figure 4a–c. A small included angle was conducive to the occurrence of slip deformation and adaptation to the intrusion of the indentation tip. Thus, the generation of ISBs inside the (110)-oriented imprint was the most intense at low temperatures and retained residual traces at RT. In comparison, the ISBs inside the (111)-oriented imprint were evident at low temperatures but entirely flattened at RT. For the (100)-oriented imprint, the pressure gradient in the radial direction was the largest, leading to a circumferential distribution state for the ISBs, which was perpendicular to the in-plane expansion direction of the indentation tip. Thus, the ISBs inside the (100)-oriented imprint were intensely distorted compared with those of the faces with other orientations. On the other hand, the crystal defects in the subsurface that occurred in the current moment were covered and distorted by newly generated defects. Unlike the shear bands under the imprints in hard, brittle materials that remain in the original deformation state [24,25], the crystal defects inside metal crystals (especially the FCC crystal) are highly interactive. Therefore, small-angle grain boundaries can only be observed beneath the final imprint boundaries in Figure 5 and Figure 7, while the inside parts of the ISBs and OSBs could not be distinguished effectively using the experimental method.

The Nix–Gao model provides an effective method for explaining the strain gradient effect in crystals, which assumes that the imprint is accommodated by circular loops of geometrically necessary dislocations (GNDs) with Burgers vectors normal to the plane of the surface [26]. However, the corresponding dislocations at the nanoscale were extremely distorted with intrusive penetration of the indentation tip and twisted with the usual statistically stored dislocations (SSDs). The complex state of the crystal defects made the TEM observations ineffective [27], and a statistical method, as well as EBSD, was adopted to characterize the distribution of GNDs [28,29]. In this work, the ISBs could be considered a microscopic form on the surface of the aggregated GNDs. The copper and aluminum data of Jeffrey et al. [29] showed that the GND density was not uniform and was mainly distributed along slip surfaces, which showed good agreement with the ISBs in this work. It could be assumed that GNDs tend to expand along the corresponding slip surface in actual crystals. Meanwhile, the ISBs and discontinuity of the GND density indicated that the generation of GNDs was not a constant process but should have involved a continuous “accumulation–release” cycle. This may be helpful for the specific application of the Nix–Gao theory in practical crystalline materials.

In previous experimental tests, subsurface lattice rotations beneath imprints in single crystals were typically used to determine the local GND structure. However, the structural relationship between the lattice rotations and GNDs remains unclear. One understanding is that small-angle boundaries divide the subsurface into several areas, where the lattice misorientation is distributed [30]. However, the most prominent small-angle boundaries were generated just beneath the boundary between the imprint and free surface, as shown in Figure 5 and Figure 7b, which was due to the large plastic deformation during the penetration process. It was difficult for the potential small-angle boundaries resulting from GNDs to compete effectively. Moreover, the continuous change in the two rotation directions on one side of the imprint could not be well explained. However, a more concise explanation can be given from another point of view, considering the plastic crystallographic slip and corresponding crystal direction.

**Figure 8:**
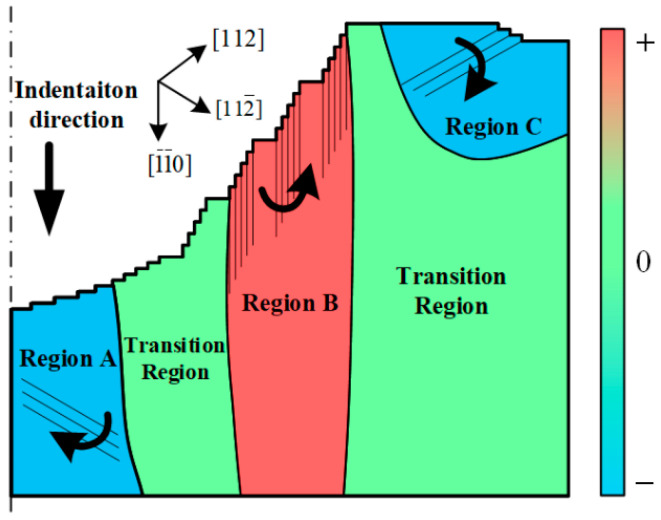
Schematic of generation of lattice rotation beneath imprint.

It can be noted that three main deformation directions could be identified during the indentation process from Figure 6. Because the Peierls–Nabarro force in FCC crystal metals is relatively small, atoms at the edge of the imprint could be extruded upward along the slip surface (region C) to the free surface, resulting in OSBs outside the indentation region. Meanwhile, more atoms inside the imprint were pushed downward, forming the height of the imprint. One direction was along [ $\bar{1}$ $\bar{1}$ 0] beneath the rim of the imprint and formed ISBs (region B), whereas the other deformation was along [11 $\bar{2}$ ] directly below the indentation tip (region A). Indentation is a penetration process in which the material undergoes limited deformation. The very closed region around and beneath the indentation tip deformed to adapt to the shape of the indentation tip, while the surrounding region tended to maintain the original crystal distribution state. Therefore, the regions where crystallographic slips occurred beneath the imprint inevitably led to lattice misorientation, as shown in Figure 8. The direction of rotation could be explicitly determined based on the relative slip direction, resulting in the misorientation being positive in region B, while regions A and C performed negatively (positive values indicate a counter-clockwise rotation). Meanwhile, two transition regions should emerge between the adjacent positive and negative regions, which contain the transition part and the original undeformed part. The current distribution and direction of the lattice orientation region coincided exactly with the EBSD results obtained by [31]. It could be further deduced that region A should expand outward continuously with the penetration process, while regions B and C move outward by adapting to the position changes of the ISBs and pile-up, respectively.

**Figure 9:**
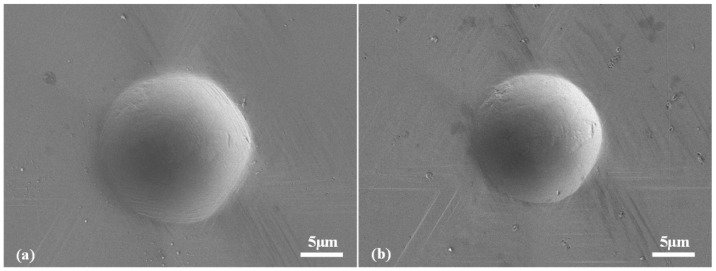
SEM images of imprints conducted under (**a**) 350 mN at 150 K and (**b**) 250 mN at RT in (111)-oriented face.

The hardness of monocrystalline copper at low temperatures was investigated in our previous work [16]. The increased hardness indicates that the ability to resist plastic deformation, which is the slip motion in monocrystalline copper, increases at low temperatures. This seems to explain the weaker pile-up and OSBs outside the imprints shown in Figure 3 at 150 K. However, the smaller size of the imprint at low temperatures may have resulted in a less obvious equality. To determine the effect of temperature, Figure 9a,b show the morphologies of imprints under 350 mN at 150 K and 250 mN at RT in the (111)-oriented face, respectively. Even though the in-plane size of the imprint conducted at low temperature was larger than that at RT, the pile-up and OSBs were still weaker. Thus, it could be concluded that low temperatures inhibited the extension of OSBs. However, we mentioned that pile-up is one of the main components of the coordinated deformation in an imprint. The plastic deformation, which should have been contributed by OSBs upward to the free surface, must have occurred downward to the subsurface. The more severe deformation demand inside the imprint may be another reason why the ISBs were more obvious at low temperatures.

### 4.2. Elastic Response

**Figure 10:**
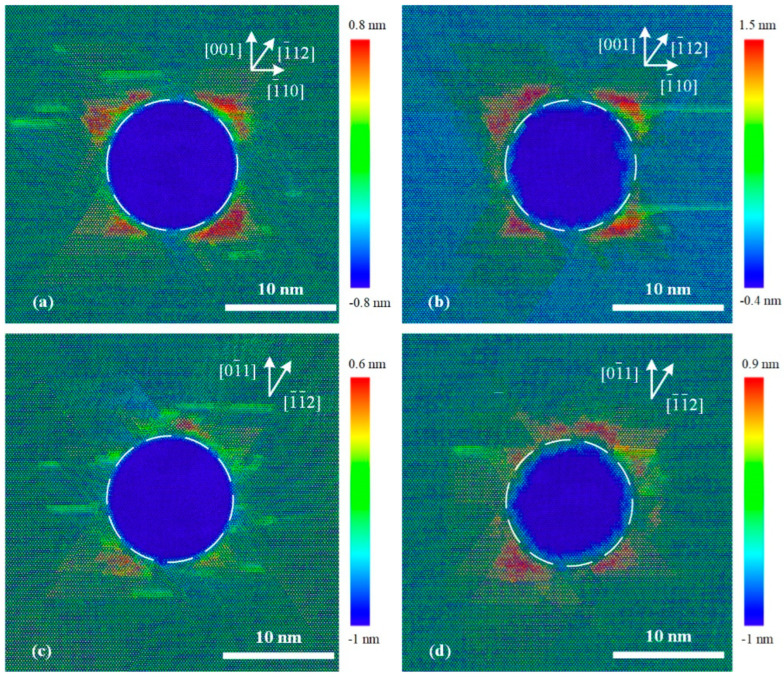
Top views of MD simulation results for imprints in (**a**,**b**) (110)- and (**c**,**d**) (111)-oriented crystal surfaces (**a**,**c**) at maximum load and (**b**,**d**) after unloading process.

Figure 10 shows the top view of the constructed MD simulation result during the unloading process for the (110)- and (111)-oriented indentations at 150 K. It can be seen that the contours of the imprints at the maximum indentation load (Figure 10a,c) exhibit nearly perfect circles in the faces with both orientations, which is the same as the cross-sectional view in Figure 6a. The high pressure applied by the indentation tip suppressed almost all the morphological features inside the imprints. The corresponding residual imprints after unloading are shown in Figure 10b,d, which exhibit anisotropic behavior. The good agreement between our simulation and experimental results in Figure 3 shows that the performance of the permanent inside imprints was due to the elastic recovery behavior during the unloading process.

**Figure 11:**
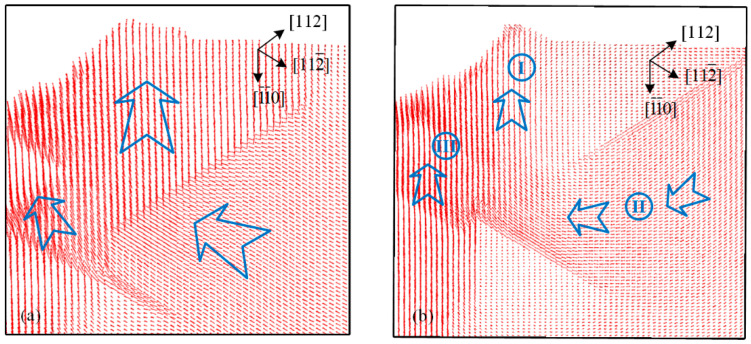
Atom trajectories of (110)-oriented indentations at (**a**) 150 K and (**b**) RT.

The atom trajectories at 150 K and RT during unloading beneath the (110)-oriented imprint are shown in Figure 11a,b, respectively. The direction of the cross-section is the same as that in Figure 6. Three regions in the main elastic recovery direction could be distinguished at both temperatures, as shown in Figure 11b. The atoms beneath the edge inside the imprint and pile-up (region I) move upward, while the range of the radial and vertical directions of the recovery atoms at 150 K is evidently wider than that at RT. The morphology of the pile-ups was inhibited by low temperatures, as seen through experimental residual imprints. The greater elastic recovery at low temperatures indicated that the generation of OSBs was even more restricted in the loading process. Meanwhile, a dividing line between regions I and II can be observed. The atoms moved along the dividing line in the region farther from the indentation center, which may have been due to the effect of slip motion stretching on the atoms underneath from the generation of OSBs during the loading process. Because the OSBs were comparatively weak at low temperatures, there was no motion component of atoms in corresponding region II along the dividing line in Figure 9a, while the upward motion trend toward the indentation center further affected the atoms in region III. The upward component of the elastic recovery motion at low temperatures was more obvious than that at RT in all the deformation regions, which was in good agreement with the elastic modulus and recovery rate of the total work calculated using the Oliver–Pharr method in our previous work [16].

**Figure 12:**
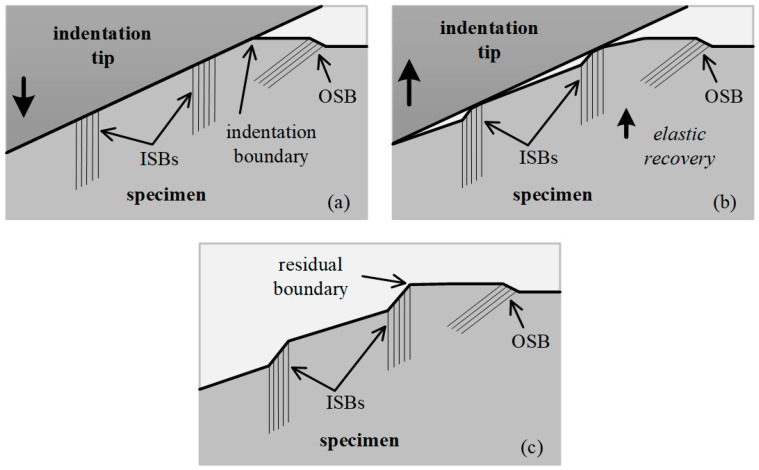
Schematics of specimen deformations (**a**) at maximum load, (**b**) during unloading, and (**c**) for residual imprint.

A schematic of the elastic recovery deformation process induced under the indentation tip is shown in Figure 12. Figure 12a shows the maximum loading state in which the ISBs and OSBs were generated during plastic deformation, whereas the surface inside the imprint adapted to the indentation tip (see Figure 6a and Figure 10a,b). Once the indentation tip was raised during the unloading process, the stored elastic deformation around the ISBs under it could be released, as illustrated in Figure 12b. In the indentation boundary region, the existing pressure was relatively small, and the elastic deformation recovered rapidly. When the indentation tip was separated from the inner surface of the imprint, the elastic recovery process around the ISBs was completed, as illustrated in Figure 12c. During the loading process, the material in the region around the indentation boundary recovered to the free surface state. The elastic recovery extended inward to the inside of the imprint until it encountered the outermost ISB, which inherited severe plastic deformation and remained as the final boundary of the residual imprint (see Figure 5 and Figure 3c,f). This behavior also occurs in “hard-to-deform directions” such as [001] in a (110)-oriented face and [1 $\bar{2}$ 1] in a (111)-oriented face and makes the corresponding boundaries indistinct without the barrier of ISBs.

**Figure 13:**
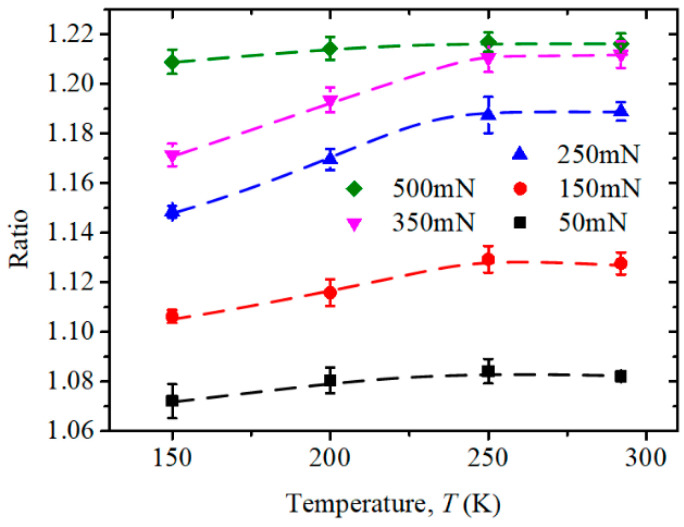
Ratio of deformable to hard-to-deform direction lengths of residual imprints on (100)-oriented face under different indentation loads at temperatures ranging from RT to 150 K.

Figure 13 shows an overview of the ratio of the deformable direction length to the hard-to-deform direction length of imprints on the (100)-oriented face, which indicates the in-plane anisotropy under different temperatures and maximum load conditions. It can be seen that the in-plane anisotropy became more obvious with an increase in the indentation load at all temperatures. This may have been due to the larger proportion of plastic deformation in the deformable directions. Meanwhile, the GND density decreased with an increase in the indentation size according to the Nix–Gao theory, resulting in fewer obstacles to the corresponding elastic recovery in hard-to-deform directions. It could also be found that the ratio decreased with a decrease in temperature under each maximum indentation load below 250 K in Figure 11. This may have been because the effect of plastic deformation in the deformable directions, as limited by low temperature, was stronger than that of elastic recovery in the hard-to-deform directions. However, it was still hard to say that the anisotropic property was weaker at low temperatures because the imprints were relatively small, and the ISB-shaped boundaries made it more complicated during elastic and plastic deformation.

## 5. Conclusions

The main results and conclusions are as follows.

1. ISBs were found inside the residual imprints of monocrystalline copper on the three oriented faces at 150 K using the experimental indentation method. The distribution of the directions of these ISBs essentially followed the active slip systems in FCC crystals but was bent because of the geometry of the indentation tip.
2. ISBs occurred at both RT and low temperatures, whereas stronger elastic recovery enabled the ISBs to be observed distinctly at low temperatures. The discontinuity of the imprint boundary at low temperatures, which consisted of indistinct and ISB-shaped states, was also due to elastic recovery during the unloading process.
3. Plastic deformation was inhibited at low temperatures, resulting in a weakened pile-up and OSBs. The material inside the imprints underwent a more downward deformation to adapt to the geometry of the indentation tip, further strengthening the phenomenon of ISBs.
4. The limited motion of the slip bands was in good agreement with the lattice rotation regions beneath the imprints. The ISBs could be considered microscopic forms on the surface of aggregated GNDs. The motions of GNDs in an actual crystal should occur along specific slip faces to match the corresponding deformations in different regions under imprints.

## Data Availability

Not applicable.
